# Increasing endogenous activity of NMDARs on GABAergic neurons increases inhibition, alters sensory processing and prevents noise-induced tinnitus

**DOI:** 10.1038/s41598-020-68652-5

**Published:** 2020-07-20

**Authors:** Di Deng, Samer Masri, Lulu Yao, Xiaoyan Ma, Xuebing Cao, Sungchil Yang, Shaowen Bao, Qiang Zhou

**Affiliations:** 1 School of Chemical Biology and Biotechnology, Peking University Shenzhen Graduate School, Shenzhen, China; 2 Department of Physiology, University of Arizona, Tucson, AZ 85724 USA; 3 South China Research Center for Acupuncture and Moxibustion, Medical College of Acu-Moxi and Rehabilitation, Guangzhou University of Chinese Medicine, Guangzhou, China; 4 Department of Biomedical Sciences, City University of Hong Kong, Kowloon, Hong Kong; 5 State Key Laboratory of Chemical Oncogenomics, Peking University Shenzhen Graduate School, Shenzhen, China

**Keywords:** Auditory system, Pharmacology, Neurological disorders, Neurophysiology

## Abstract

Selective enhancement of GABAergic inhibition is thought to impact many vital brain functions and interferes with the genesis and/or progression of numerous brain disorders. Here, we show that selectively increasing NMDA receptor activity in inhibitory neurons using an NMDAR positive allosteric modulator (PAM) elevates spiking activity of inhibitory neurons in vitro and in vivo. In vivo infusion of PAM increases spontaneous and sound-evoked spiking in inhibitory and decreases spiking in excitatory neurons, and increases signal-to-noise ratio in the primary auditory cortex. In addition, PAM infusion prior to noise trauma prevents the occurrence of tinnitus and reduction in GABAergic inhibition. These results reveal that selectively enhancing endogenous NMDAR activity on the GABAergic neurons can effectively enhance inhibitory activity and alter excitatory–inhibitory balance, and may be useful for preventing diseases that involve reduced inhibition as the major cause.

## Introduction

A proper balance between excitatory and inhibitory activity is critical to many fundamental properties of the brain, from processing of sensory information to oscillatory activity underlying working memory^1–5^. Altered inhibition (often reduced) has been proposed to contribute to the pathogenesis of various brain disorders, such as schizophrenia, autism and Alzheimer’s disease^6–8^. A better understanding of how these reductions can be ameliorated in a therapeutically feasible manner is thus of critical importance.

We have characterized small molecule positive allosteric modulators of *N*-methyl-d-aspartate receptors (NMDAR-PAMs) that enhance NMDAR activity in a glutamate [NMDA] receptor subtype 2A (GluN2A)-specific manner^9^. These PAMs only potentiate responses of active NMDARs. One of the PAMs, GNE-8324, is of particular interest since it selectively enhances activity of synaptic NMDARs on inhibitory but not excitatory neurons^9,10^. This novel selectivity is mediated by higher ambient glutamate concentration in the synaptic cleft during resting (non-stimulating) conditions in inhibitory neurons^10^. However, whether this selective enhancement translates to a selective increase in inhibitory neuronal activity in vivo is unknown. Furthermore, its potential therapeutic applications have not been explored. In this study, we have addressed these questions by examining the in vivo effects of an improved version of GNE-8324 (termed M-8324) which showed similar properties and selectivity as GNE-8324.

For sensory processing, inhibition makes significant contributions to the reliability of signal transformation, signal-to-noise ratio (SNR), receptive field properties, and gamma oscillations^11–14^. Reduced response reliability (increased variability) and SNR have been shown in schizophrenia and autism patients and animal models^15–18^. These findings point to the importance of balanced excitation and inhibition, which is observed for both sensory-driven and spontaneous activity^19^. For long-term brain function and health, reduced inhibition may trigger long-lasting changes in circuitry and neuronal function, and, in extreme cases, neural loss. For example, reduced inhibition has been reported in the early stage of tinnitus and is proposed to contribute to its genesis and progression^20^, and decreasedγ-Aminobutyric acid (GABA) concentration has been reported in patients with hearing loss^21^.

Enhancing inhibition by elevating the activity of GABAergic neurons has been achieved via optogenetic or chemogenetic methods. While important in addressing the functions of specific GABAergic subpopulations, these approaches have a few limitations: (1) activation is usually unnaturally strong with synchronized activation of many neurons which does not reflect physiological patterns; (2) activation is usually limited to one subtype of inhibitory neurons. Endogenous behavior likely requires coordinated activation of many subtypes of GABAergic neurons^14^, and modulation of diverse GABAergic neurons may be needed or required to achieve therapeutic benefit since various subtypes are altered in diseases^22,23^; (3) these approaches are not ready for clinical use yet. By using small molecule NMDAR- PAMs, we circumvent the above limitations, since PAMs enhance endogenous activity rather than imposing activity artificially, and likely affect the majority of inhibitory neurons^10^.

Here, we report that in vivo infusion of M-8324 enhanced both spontaneous and sound-evoked spiking in inhibitory neurons while reducing the activity of excitatory neurons in the auditory cortex. These changes are associated with enhanced SNR for sound- evoked responses, while reducing the reliability of information transfer. Infusing M-8324 prior to noise exposure prevents the occurrence of tinnitus and partially ameliorates reduction in GABAergic functions/markers associated with tinnitus.

## Results

### M-8324 selectively enhances synaptic NMDARs on inhibitory neurons and promotes their spiking in vitro

**Figure 1:**
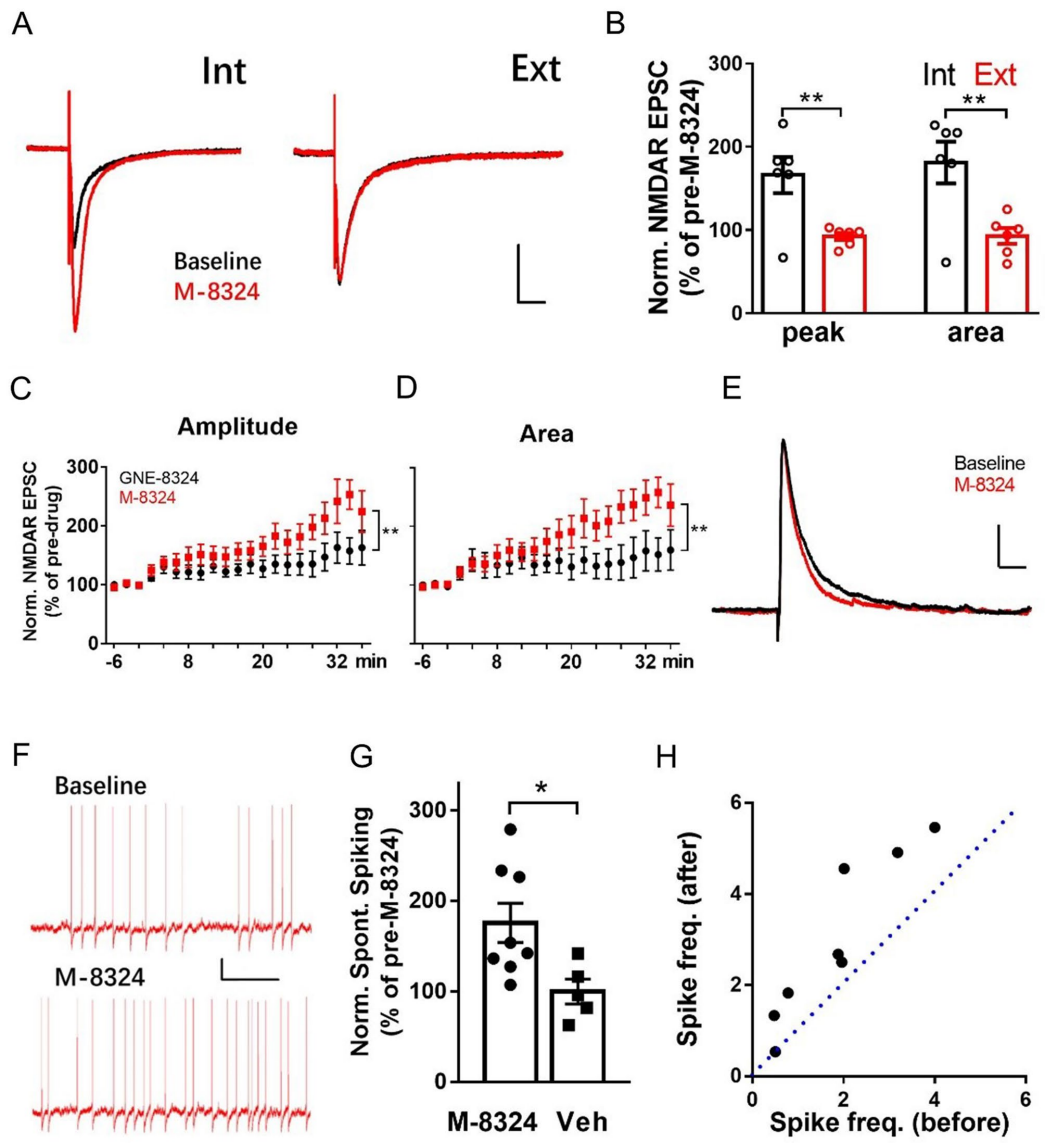
M-8324 enhances spontaneous spiking of GABAergic neurons in vitro. (**A**) Sample traces showing 30 µM M-8324’s effect on NMDAR EPSCs in inhibitory neurons (Int) and excitatory neurons (Ext). Scale bars, 100 ms, 50 pA. (**B**) Population results on normalized EPSC peak and area during bath application of M-8324 (30 µM) for Ext and Int neurons. N = 6 cells/3 mice for each group. Normalized isolated NMDAR EPSC amplitude (**C**) and area (**D**) before and after bath perfusion of GNE-8324 or M-8324 in GABAergic neurons. N = 12 cells/4 mice for GNE-8324, N = 11 cells/4 mice for M-8324. (**E**) Sample recording showing no impact of M-8324 on AMPAR-EPSPs in GABAergic neurons. Scale bars, 50 ms, 2 mV. (**F**) Sample voltage traces showing M-8324′s effect on spontaneous spiking in GABAergic neurons in whole-cell recording mode. Scale bars, 4 s, 10 mV. (**G**) Normalized spontaneous spiking frequency during either M-8324 or Veh bath application in GABAergic neurons in AI slice. N = 8, 5 cells/3 mice for M-8324 and Veh, unpaired *t* test. (**H**) Frequency of spontaneous spiking before and after M-8324 application in the same neurons. Dotted line represent slope of 1 (no change). Data are presented as the mean ± SEM.

We first confirmed that a modified version of GNE-8324 (M-8324) also showed a selective enhancement of synaptic NMDAR responses in inhibitory but not excitatory neurons (Fig. 1A, B). In addition, M-8324 showed larger potentiation than GNE-8324 at the same concentration tested (Fig. 1C, D), suggesting higher potency. We have further tested M-8324 on α-amino-3-hydroxy-5-methylisoxazole-4-propionic acid (AMPA) receptor-mediated excitatory postsynaptic potential (EPSPs) in the GABAergic neurons and found no effect (Fig. 1E), supporting its selectivity for NMDARs. The gradual increase in the NMDAR responses is likely due to a gradual building-up of M-8324 at the recorded neurons, which is typical for drugs we have used in other experiments. It is possible that lateral movement of NMDAR could be altered by M-8324, although we think this possibility is remote. Since GNE-8324 potentiates both synaptic and extrasynaptic NMDAR responses^10^, it is unlikely that the impact of NMDAR-PAMs on the NMDAR trafficking plays a major role since it is unlikely that the same drug enhances NMDAR trafficking to both synaptic and extrasynaptic sites.

As a first step to test M-8324 effect on neural spiking, we recorded spontaneous spiking in inhibitory neurons (in GAD_67_-GFP knock in mice) in layer2/3 of primary auditory cortex (AI) using whole cell recording. Bath perfusion of M-8324 (30 µM) led to a significant increase in spike frequency, which was absent in vehicle-treated neurons (Fig. 1F, G). There was a rough linear relationship between spike rate before and after M-8324 application (Fig. 1H), consistent with potentiation of M-8234 on spiking being proportional to the endogenous activity level rather than clamping spiking to the same level. In some GABAergic neurons, spontaneous spiking was seen in the cell-attached mode and this spiking was enhanced by bath perfusion of M-8324 (data not shown).

### M-8324 enhances spiking in inhibitory neurons and alters balance between excitation and inhibition in vivo

**Figure 2:**
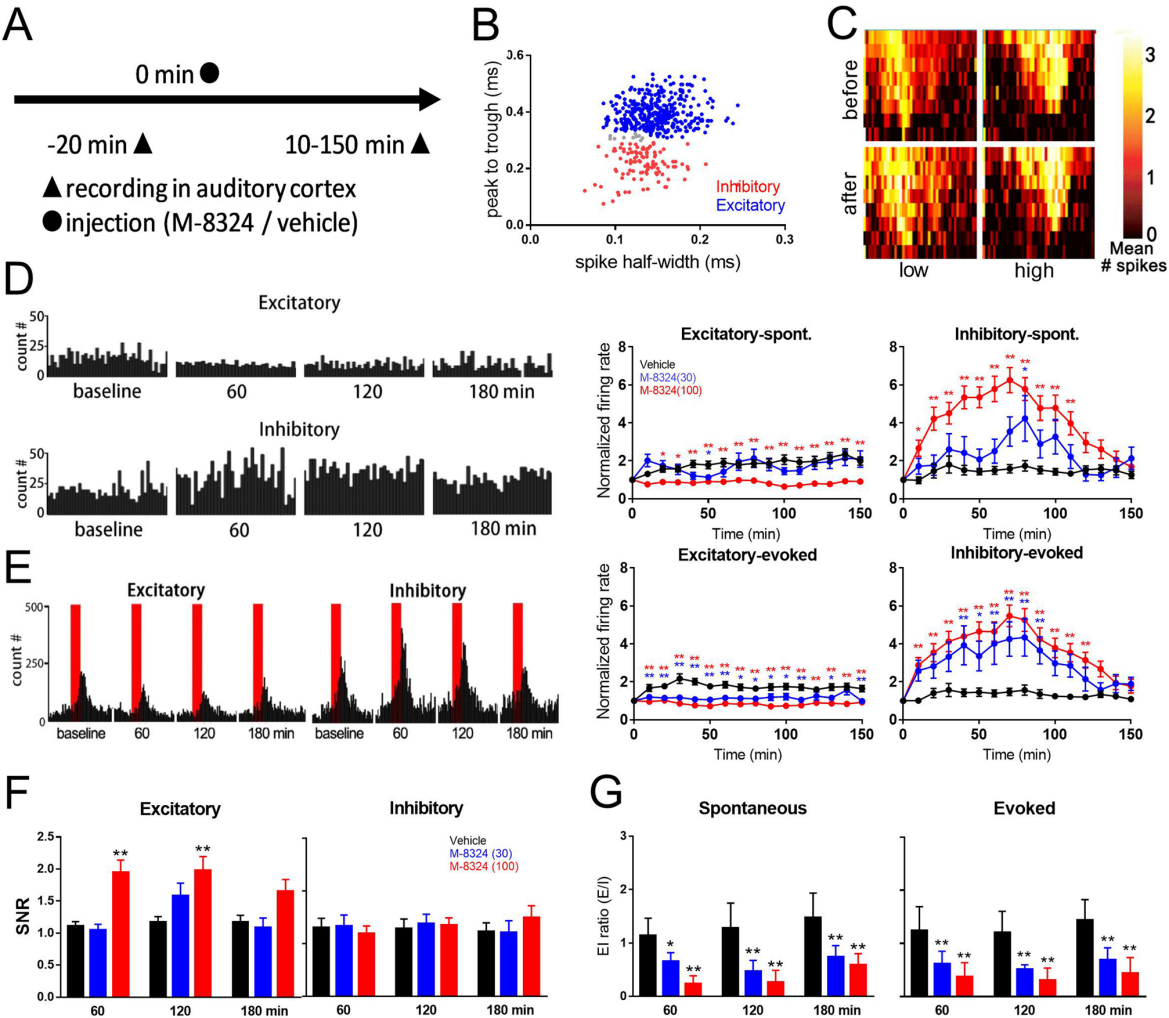
M-8324 altered rates of sound-evoked and spontaneous spike in AI in vivo. (**A**) Experimental procedures. (**B**) Identification of putative excitatory (red) and inhibitory (blue) neurons based on their spike waveforms, while light gray dots between the blue and red dots were excluded from data analysis. (**C**) Example receptive fields during “before drug” (upper) and “after drug” (lower) trials for the same recording site in AI. Each pixel in the plot represents the average number of spikes evoked by a sound stimulus at a particular frequency and intensity level. (**D**) (Left) Examples of an increase in the frequency of spontaneous spiking in inhibitory neurons concomitant with a decrease in excitatory neurons after 100 µM M-8324 infusion. (Right) Population results and time course of changes in spontaneous spiking in excitatory and inhibitory neurons. (**E**) (Left) An increase in the sound-evoked spike frequency in inhibitory neurons concomitant with a decrease in excitatory neurons after 100 µM M-8324 infusion. (Right) Population and time course of changes. Neurons were the same as in (**D**). (**F**) A significant increase of SNR in excitatory neurons but no change for inhibitory neurons. (**G**) A significant increase in the E/I ratio for both spontaneous spiking (left) and sound-evoked responses (right). For excitatory cells, N = 95 cells/8 mice (vehicle), 73 cells/6 mice (M-8324 30 µM), 114 cells/9 mice (M-8324 100 µM). For inhibitory neurons, N = 28 cells/8 mice (vehicle), 19 cells/6 mice (M-8324 30 µM), 46 cells/9 mice (M-8324 100 µM). Data are presented as the mean ± SEM. * represent significance versus vehicle group.

We next asked whether M-8324 can also increase spiking in inhibitory neurons in vivo*,* and if so whether this enhancement also occurs with natural inputs. To do so, we recorded from AI to examine both spontaneous spiking and sound-evoked spiking. We have used two anesthetics for these experiments (see “Methods” section), since these results were qualitatively similar, we have presented the pooled results. We recorded neural activity using multi-electrode arrays in AI in anesthetized mice and tested two different concentrations of M-8324 (Fig. 2A). Based on the spike waveforms, we separated the recorded neurons into regular spiking (RS) and fast spiking (FS), and based on previous studies, it is generally agreed that RS neurons are mostly consisted of excitatory neurons while FS neurons are mostly parvalbumin (PV)-positive inhibitory neurons (Fig. 2B; see “Methods” section). Hence, for the purpose of simplification, in the subsequent text, we called RS neurons excitatory and FS inhibitory neurons.

Tuning curve properties were not significantly affected by M-8324 (Fig. 2C, Fig. S1, S2). A significant increase in the frequency of spontaneous and sound-evoked spiking in inhibitory neurons was concomitant with a significant reduction of those spiking in excitatory neurons (these changes started at 20 min after M-8324 infusion into brain ventricle and lasted longer than 90 min; Fig. 2D, E). In the recorded neuronal population, about 30% did not respond to sound, but these neurons also showed similar changes to M-8324 (Fig. S3), and they were not characterized further. Thus, M-8324 can enhance both spontaneous and sound-evoked spiking in inhibitory neurons in vivo.

Previous studies have shown that changes in inhibition level may alter SNR and E/I ratio for the transmitted information (such as sound). We thus plotted SNR defined as evoked spike frequency over spontaneous spike frequency, and we found a significant increase for the excitatory neurons at 60 min and 120 min (Fig. 2F). Interestingly, there was no change for inhibitory neurons at all three time points and all M-8324 concentrations tested (Fig. 2F). As expected, E/I ratio was reduced for all three time points in both spontaneous and sound evoked spiking rate, by computing spike frequency of excitatory neurons over that of inhibitory neurons (Fig. 2G). Together, this increased SNR in AI after M-8324 infusion is likely caused by enhanced inhibition.

**Figure 3:**
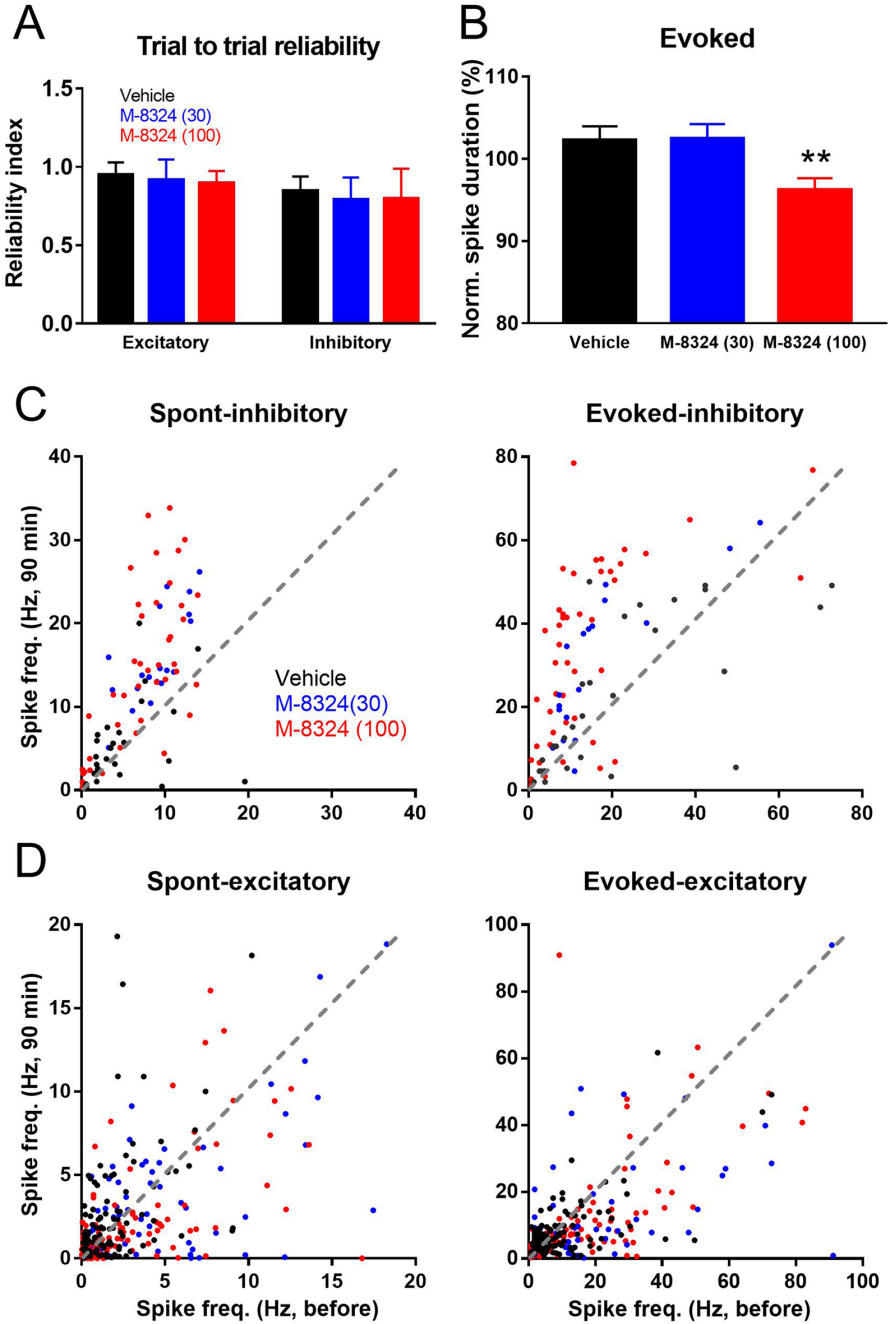
M-8324’s effect on trial to trial reliability and duration of sound evoked spiking. (**A**) Trial to trial reliability of sound evoked responses were not altered in the presence of 100 µM M-8324 (between 90 and 100 min after infusion), in both excitatory and inhibitory neurons. For excitatory neurons, N = 8 mice (vehicle), N = 6 mice (M-8324 30 µM), N = 9 mice (M-8324 100 µM). (**B**) A significant reduction in the duration of evoked responses in excitatory neurons by 100 µM M-8324. (**C**, **D**) Effect of M-8324 on spontaneous spiking (**C**, left) and sound-evoked spiking (**C**, right) in inhibitory neurons, spontaneous spiking (**D**, left) and sound-evoked spiking (**D**, right) in excitatory neurons. Dotted lines are of slope of 1 (no change). Data are presented as the mean ± SEM. * represent significance compared to vehicle group.

To understand whether the fidelity of signal transmission is affected by M-8324, we measured trial-to-trial reliability of sound-evoked response between 90 and 100 min after M-8324 infusion using Pearson correlation. We found that reliability was not significantly altered for either cell type or at both M-8324 concentrations (Fig. 3A), suggesting that reliability is largely preserved in the presence of M-8324.

How can enhanced activity of inhibitory neurons lead to a larger reduction in spontaneous spiking as opposed to evoked spiking in excitatory neurons? Since the majority of recorded AI inhibitory neurons provide either feedback or feedforward inhibition to excitatory neurons during transmission of sensory information^24^, there are likely certain constraints on the effectiveness of these inhibitory neurons. For example, a difference of a few milliseconds, corresponding to one synaptic delay, between the arrival of excitatory and inhibitory transmission at the target/recorded neurons may mean that enhanced IPSPs (due to enhanced spiking of inhibitory neurons) cannot impact EPSPs and spiking prior to IPSP occurrence^25,26^. It is thus predicted the duration of spiking in excitatory neurons to be reduced, but not the latency to spiking from sound onset, in the presence of M-8324. Supporting this prediction, we found a significant reduction in the duration of evoked spiking in excitatory neurons following infusion of 100 µM M-8324 (Fig. 3B), but the delay to first spike was not affected (Fig. S4). This suggests that later spiking during sound presentation was reduced or eliminated by enhanced inhibition in the presence of M-8324.

As stated in the introduction, one likely difference between PAMs and optogenetic stimulation is that PAMs may preserve endogenous activity patterns. To examine whether this is the case for M-8324 in vivo, we plotted spike frequency before and after infusion of M-8324. For inhibitory neurons, most of the data points fell above the dotted line (slope of 1) consistent with increased spike rate in the presence of M-8324, for both spontaneous and evoked responses (Fig. 3C). More importantly, for most neurons, the increase is roughly proportional to the initial (pre-drug) spike frequency, and there was no indication of M-8324 clamping spiking to the same level, consistent with endogenous activity patterns being roughly preserved. A similar effect was present for excitatory neurons but less striking (Fig. 3D).

### M-8324 infusion prevents tinnitus and reduced GABAergic functions induced by noise exposure

To address whether M-8324 can prevent the genesis of brain diseases caused by reduced inhibition, we decided to examine whether tinnitus can be prevented by infusion of M-8324 prior to its induction by a noise-induced hearing lesion based on two reasons: (1) the onset of tinnitus occurs rapidly after noise exposure (within 10 days)^27,28^ and hence may be effectively modulated by a short-term drug infusion; (2) reduced GABAergic function has been found to be associated with tinnitus induction, and interference with this reduction prevents tinnitus occurrence^27,28^.

**Figure 4:**
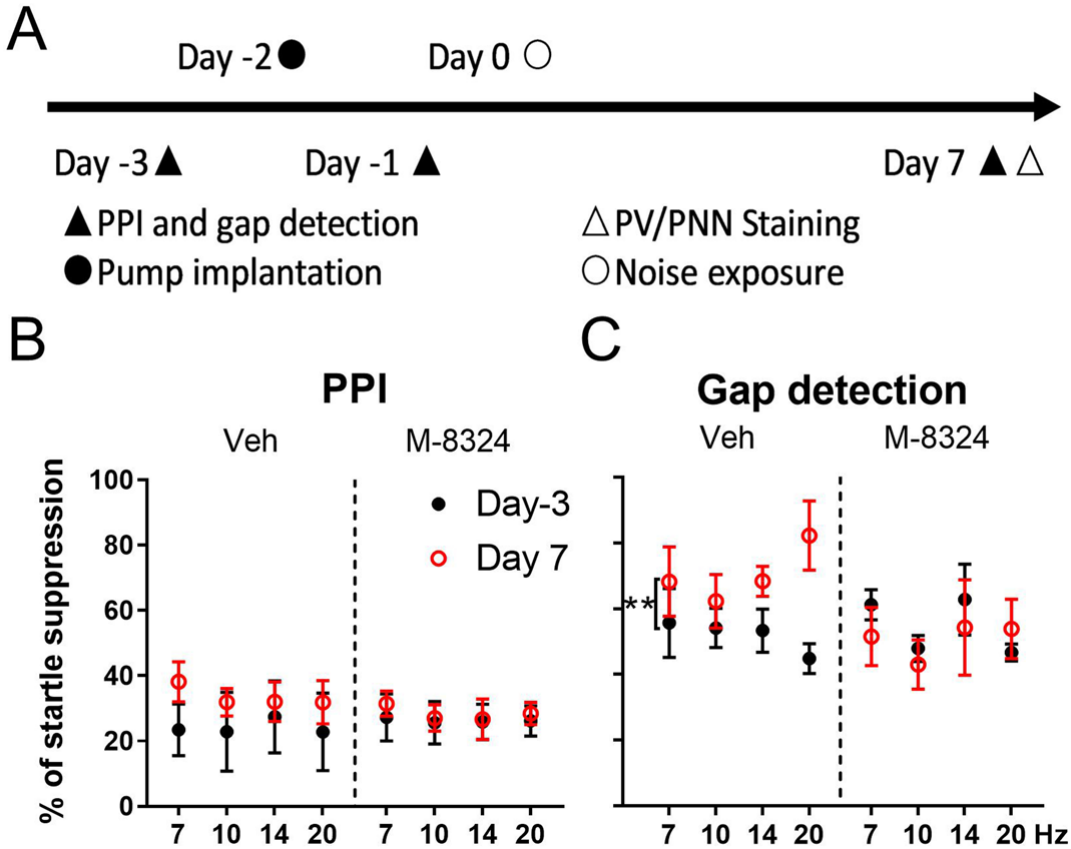
Infusion of M-8324 prior to noise exposure prevented tinnitus in noise exposure model. (**A**) Experimental procedure. (**B**) Pre-pulse inhibition was not altered by M-8324, compared with Veh, in noise exposure model. N = 5 mice for each group. (**C**) M-8324 prevented deficit in gap detection in noise exposure model. Percent of startle suppression in M-8324 group was significantly lower than that in the Veh group. Data are presented as the mean ± SEM.

Exposure to loud noise of a given frequency (8 kHz, 115 dB for 2 h) led to symptoms of tinnitus within 10 days, as measured using pre-pulse inhibition (PPI) and gap detection (Fig. 4A). There was no difference in PPI measurements, between 3 days prior to and 7 days after noise exposure, for either M-8324 or veh-infused group (Fig. 4B), suggesting that hearing deficits are not altered by noise exposure or drug infusion^29^. In contrast, a significant difference was seen in startle response ratio during a gap detection test in the veh-group between 3 days prior to and 7 days after noise exposure, while no difference was seen in the M-8324 group (Fig. 4C). Since deficits in gap detection are usually used to infer the occurrence of tinnitus, the above results suggest that M-8324 infusion prevents tinnitus.

**Figure 5:**
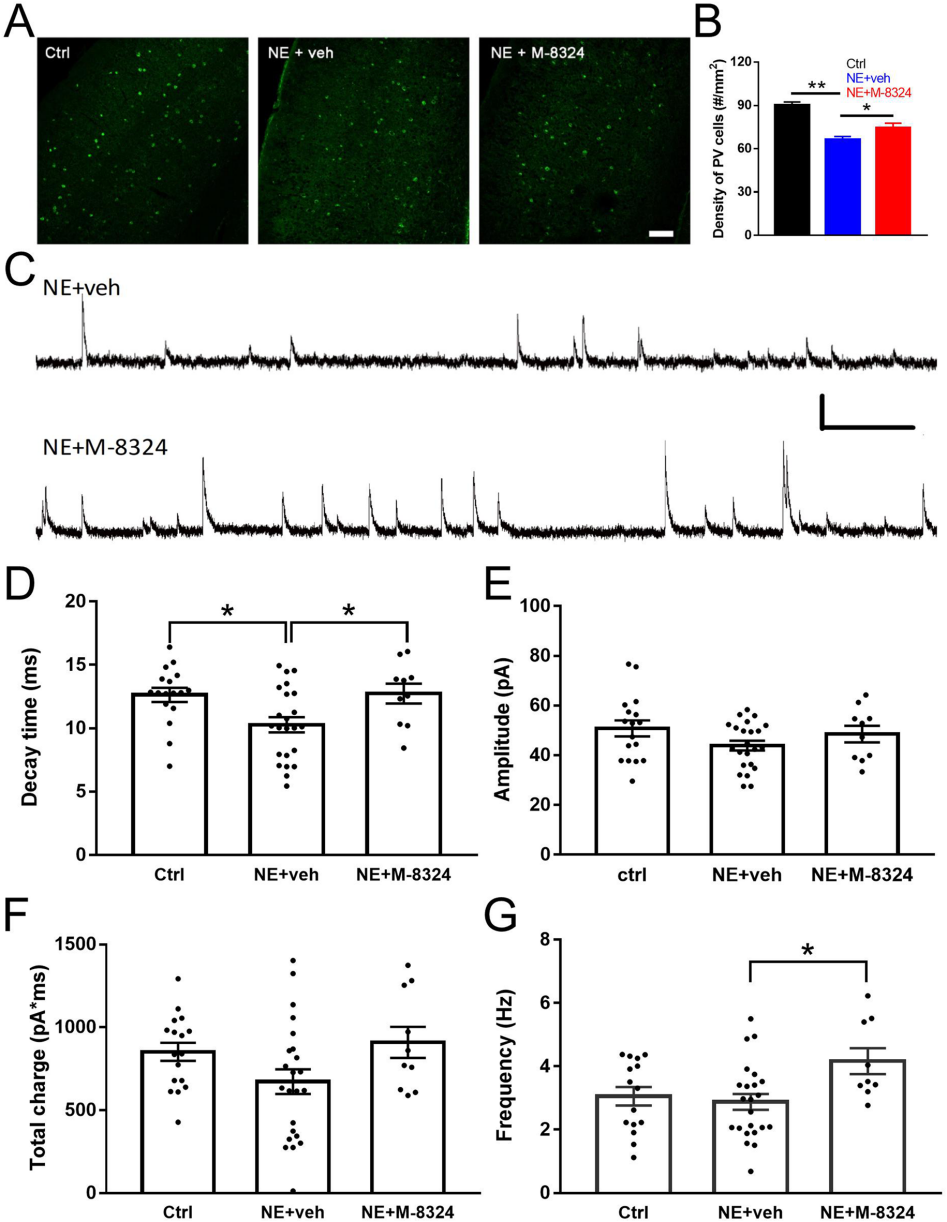
M-8324 infusion prior to noise exposure reduced PV-neuron loss and increased inhibitory synaptic transmission in AI in the noise-exposure (NE) mice. (**A**) Representative images of staining of PV-neurons in AI of control, NE + veh and NE + M-8324 group. Scale bar, 100 µm. (**B**) Density of PV-neurons in AI layer 2/3 in. N = 30 sections/5 mice for Ctrl group, N = 22 sections/5 mice for NE + veh group, N = 15 sections/4 mice for NE + M-8324 group (**C**) Sample traces of spontaneous inhibitory postsynaptic current (sIPSC) from AI excitatory neurons in NE + veh group and NE + M-8324 group. Scale bars, 40 pA/500 ms. (**D**) A significant reduction in the decay time of sIPSCs in NE group. (**E**) Amplitude of sIPSC in different among groups. (**F**) Charge of sIPSC in different among groups. (**G**) A significant increase in the frequency of sIPSC was seen in NE + M-8324 group. N = 17 cells/4 mice for Ctrl group, N = 23 cells/4 mcie for NE + veh, N = 10 cells/4 mice for NE + M-8324. Data are presented as the mean ± SEM.

One likely underlying mechanism for the above prevention of tinnitus is that M-8324 effectively elevates GABAergic function at the onset of tinnitus to prevent or counteract noise exposure-induced reduction in GABAergic transmission. We thus examined whether M-8324 infusion affects GABAergic transmission after noise exposure. First, the density of PV-expressing neurons in layer 2/3 of AI showed a significant reduction in noise-exposure mice compared to naïve mice, and this reduction was significantly smaller in mice infused with M-8324 (Fig. 5A, B). Second, we recorded sIPSC in excitatory neurons in AI (Fig. 5C), and found a significant reduction in the decay time in noise-exposed mice which was reversed in the M-8324 group (Fig. 5D), but there was no significant change in either amplitude (Fig. 5E) or total charge (Fig. 5F). Interestingly, a significant increase in the frequency of sIPSC was also seen in mice infused with M-8324 (Fig. 5G), consistent with an increased presynaptic activity-driven enhancement in inhibition by M-8324 which may prevent the occurrence of tinnitus.

## Discussion

In this study, we found that M-8324 (modified version of GNE-8324), a NMDAR-PAM that selectively enhances NMDAR responses on inhibitory neurons, increases both spontaneous and sound-evoked spiking in inhibitory neurons. This enhanced inhibition is associated with reduced spontaneous and sensory-evoked spiking in excitatory neurons, and improved SNR for sound-evoked signals. Furthermore, infusion of GNE-8324 prior to noise exposure prevents the occurrence of tinnitus and associated reduced GABAergic synaptic transmission and density of PV neurons. These findings indicate that enhanced endogenous inhibition may modulate sensory information processing and prevent disease genesis.

Higher potency was seen in M-8324 compared to GNE-8324, while the selectivity for synaptic NMDAR responses in inhibitory neurons is retained for M-8324. There was also no effect of M-8324 on AMPAR-mediated EPSPs in the GABAergic neurons. Whether M-8324 affects only GluN2A-NMDARs, as is the case for GNE-8324^9,10^, was not tested. In addition to neurons in AI, we have also found enhanced NMDAR-EPSCs/EPSPs and spiking in GABAergic neurons in mouse prefrontal cortex and hippocampus, suggesting that this enhancement is likely brain-wide. Although we have not systematically tested M-8324 on the various subtypes of identified GABAergic neurons (such as those containing PV or somatostatin (SOM) using transgenic mice), we have seen efficacy (both NMDAR-EPSCs and spiking) in the majority of GABAergic neurons that we have recorded in brain slices. Hence, the effect of M-8324 is unlikely restricted to a subtype of subpopulation of GABAergic neurons.

### Enhanced inhibition on evoked and spontaneous activities

M-8324 shows differential impacts on excitatory and inhibitory neurons: sensory-evoked and spontaneous spike rates were similarly enhanced in the inhibitory neurons while the reduction is larger for spontaneous than for sound-evoked spiking in the excitatory neurons. This differential change underlies the observed increase in SNR. This is somewhat surprising since a well-balanced E–I ratio has been reported for both spontaneous and evoked activity in normal animals^30,31^. During sound-evoked responses, feed-forward inhibition suppresses excitation with a mono-synaptic delay and its enhancement by M-8324 mainly cuts short the duration of spiking in excitatory neurons (Fig. 3B). In contrast, suppression of spontaneous spiking in excitatory neurons by elevated inhibitory neuron spiking is not limited by this anatomical restriction and results in a larger reduction^32–34^. Alternatively, NMDARs could have different contributions to stimulus-driven versus spontaneous activity in the excitatory vesus inhibitory neurons. This scenario is complex and requires further examination.

These findings on neuronal spiking are consistent with M-8324 directly enhancing GABAergic neuronal activity as we have shown in cortical slices, and the enhanced GABAergic neuronal activity in turn reduces spiking of excitatory neurons that they innervate. Since M-8324 was infused into the brain ventricle, the entire brain is bathed in M-8324 during drug application which should affect inhibitory neurons and excitatory neurons throughout the brain, including subcortical regions which provide inputs to AI. The end effect of these complex interactions (between subcortical and cortical areas, different layers within the cortex, etc.) cannot be easily computed from recordings in the related individual regions/areas participating in this circuitry. This complexity may be contributed by differences in NMDAR composition (for example, GluN2A-diheteromeric receptors vs. GluN2A-triheteromeric receptors) and the degree of NMDAR activation which may differ among different brain regions and specific brain states. This question may be addressed by measuring brain activity at a much larger scale, using MRI and PET. Since we are at the beginning of understanding the efficacy and potential therapeutic values of these NMDAR-PAMs in vivo, what they do and can do is more important than how they do them at this moment. Nonetheless, a more thorough understanding of the issues is critical to advance our understanding of the contributions of NMDARs to brain functions and diseases in general.

In the dorsal cochlear nucleus, reduced spontaneous spiking of inhibitory neurons is associated with enhanced evoked transmission, and an enhanced SNR for inhibitory signal transmission^35^. In the hippocampus CA1, seortonin agonist and cholecystokinin (CCK) increase the spontaneous spiking of PV-neurons which suppresses the spontaneous spiking of their targeted excitatory neurons^25,35^. Due to use-dependent short-term depression of GABA release from PV-neurons, this enhanced spontaneous spiking of PV-neurons results in reduced action-potential-evoked transmission onto excitatory neurons and enhanced spiking fidelity in excitatory neurons. We previously showed that optogenetic activation of PV-neurons reduced spiking in AI in general while enhancing the functional connectivity^24^. Duguid et al. found that enhancing tonic inhibition via enhancing GABA_A_R activity reduces spiking probability and SNR of sensory information without significantly affecting spontaneous activity level in the cerebellar cortical output neurons^36^. We found that SNR is enhanced in the excitatory neurons but not inhibitory neurons, suggesting that modulation of SNR might be distinct between excitatory and inhibitory neurons. This finding is similar to that of Ma et al.^37^ in monkeys with ketamine. Two studies showed that reduced activity of a specific subtype of GABAergic cell population (such as PV-neurons) led to reduction in both SNR and signal reliability^13,15^. One possible explanation of this discrepancy with our current finding is that those studies targeted a selective subpopulation while we targeted a majority of GABAergic neurons, whether targeting a few subgroups and any GABAergic neurons simultaneously may affect signal reliability in a different way is worthy of further examination. Put together, altering basal inhibition level show similar and consistent impact on the basal spiking of excitatory neurons, but its impact on evoked spiking in the excitatory neurons and SNR may depend on the specifics of the alterations (such as on which side of the synapses modulation occurs and the level of modulation).

One possible explanation for the discrepancy discussed above is that activation of PV neurons by M-8324 is not as strong or synchronized as by optogenetic stimulation, and hence may not lead to significant depression to affect evoked-release. Another possibility is the specific inhibitory neuron populations being modulated by M-8324 might be different. From our previous work^9,10^, we have not seen a significant difference among the GABAergic neurons (GAD_67_-GFP) in response to GNE-8324, and hence it is likely that M-8324 may affect most GABAergic neurons. Since we can only identify FS neurons in our in vivo recordings as likely inhibitory neurons, it remains to be tested directly whether non-FS/PV GABAergic neurons behave in a similar way in the presence of M-8324 in vivo. Further characterization of the exact inhibitory neuron subtypes activated by NMDAR-PAMs (GNE-8234, M-8324, etc.) and the underlying mechanism (such as the presence of NMDARs on these neurons) will be of importance.

### Enhanced inhibition may be therapeutically valuable

For tinnitus and likely other trauma-related brain diseases, an initial reduction in GABAergic transmission occurs prior to symptom onset^38^. In noise exposure-induced tinnitus, reduced IPSCs in AI excitatory neurons, GABAergic and glycinergic neurotransmission in inhibitory neurons, as well as PV-neuron density, have been reported^39–44^. Whether this reduced density is caused by loss of PV-neurons or reduced PV expression is still an open question that needs further experimentation. One scenario is that reduced GABAergic signaling leads to hyperactivity and eventual loss of PV-neurons. Infusion of M-8324 prior to noise exposure could partially counteract these changes in GABAergic signaling. Interestingly, in addition to reversing/preventing noise-induced reduction in the decay of sIPSCs, an increase in the frequency of sIPSCs was also found in M-8324 treated mice, consistent with M-8324 acting presynaptically to enhance spiking of GABAergic neurons.

Elevated spontaneous activity (likely resembling noise) has been reported in schizophrenia and aging, and has been proposed to be caused by reduced inhibition^45,46^. In aged rats, extensive sensory training rescued functional deficits concomitant with increased density of PV-neurons^47^. Thus, M-8324 might be useful for reducing the impact of aging by slowing down the natural decay in inhibitory functions during aging. Reduced inhibition is proposed to reduce the reliability of information transmission and/or processing, seen as reduced response reliability and SNR in human patients and animal models of schizophrenia and autism^15,17,18,48^ . Although improved SNR could be caused directly by enhanced NMDAR activity since previous studies showed that administering NMDAR antagonists reduced SNR^49^, these reported effects could be mediated by altered inhibition by NMDAR antagonists/PAMs acting on inhibitory neurons^50–52^. One potential advantage of enhancing both spontaneous and evoked inhibition, as opposed to enhancing one but reducing the other (as seen in some studies), is to prevent seizure genesis. Reduced inhibition, for tonic/spontaneous or inputs-evoked, especially with a substantial magnitude and prolonged period of time, is likely to elevate the risk of seizure.

The effects of M-8324 on sensory transmission and processing have commonalities and differences from those reported through optogenetic activation of GABAergic neurons. For example, Zhu et al.^13^ showed that suppressing PV-neurons is sufficient to increase responses to visual stimuli and improves SNR. Manipulating PV neuron activity also alters the spontaneous activity level in the excitatory neurons^13,14,24,53–62^. Our recent study showed that deficit in gap detection associated with noise exposure-induced tinnitus is mostly mediated by reduction in PV- but not SOM-neurons, and activation of PV-neurons using DREADD can partially ameliorate this deficit^63^. One distinct feature of M-8324/NMDAR-PAMs vs. optogenetic activation is preservation of endogenous activity patterns in that enhancement is roughly proportional to basal activity levels (pre-drug level) (Fig. 3C, D). For therapeutic applications, enhancement of endogenous firing patterns will help alleviate symptoms while preserving natural activity patterns associated with highly coordinated behavioral states.

### Unresolved questions

It remains to be tested that M-8324 might affect non fast-spiking/PV neurons and other functions mediated by these GABAergic neurons which we did not examine. The above “benefits” mediated by M-8324 are likely to be greater in disease states/animals where there is a deviation from the norm. It is also possible that this initial reduction in GABAergic signaling might take some time to develop since it may likely to be homeostatic in nature and occurs over a protracted period of time. It is then possible that if enhancing inhibition (with M-8324) occurs soon after noise exposure, tinnitus might be prevented. In addition, there are many aspects of long-term enhancement of inhibition needs to be explored. For example, is a new persistent E/I balance achieved after washout of M-8324?

From results of ours and others, it is evident that enhanced inhibition from either presynaptic or postsynaptic side is sufficient to compensate for the reduced GABAergic system to interfere with tinnitus^64,65^. Given the addiction potential and other side-effects of benzodiazepam type drug, enhancing inhibitory effects using M-8324 is likely more therapeutically appealing. Interestingly, both benzodiazepam and M-8324 are PAMs.

### Conclusions

In this study, we provided clear evidence that a small molecule NMDAR-PAM, M-8324, increases spiking of GABAergic neurons, alters sensory processing and prevents noise exposure-induced tinnitus. The differential impacts of M-8324 on evoked vs. spontaneous activity is worthy of further investigation to determine whether it is a general feature of increased inhibition or specific to M-8324. In addition, the efficacy, limitations and therapeutic applications of enhancing inhibition, especially at an early stage of disease progression, warrants further research to fully understand its therapeutic potential.

## Methods

### Animals and experimental design

All experimental procedures were approved by Peking University Shenzhen Graduate School Institutional Animal Care and Use Committee (IACUC) (Approval Number: AP0011002) and the University of Arizona IACUC (Approval Number: 14-554). Research protocols were consistent with the guidelines and regulations of Good Laboratory Practice for non-clinical laboratory studies of drugs issued by the National Scientific and Technological Committee of PRC and Good Laboratory Practice for Nonclinical Laboratory Studies issued by USFDA.

C57BL/6 J mice (stock # 000664) were purchased from Medical Laboratory Animal Center (Guangdong, China) and Jackson Laboratory (Bar Harbor, ME, USA). GAD67-GFP knock-in mice on CD1 background obtained from Dr. Miao He (Institutes of Brain Science, Fudan University) were used for identifying and labeling GAD positive inhibitory neurons (including calretinin, PV and SOM)^66^ for experiments shown in Figs. 1 and 5C–G. All mice were randomized for treatment. In cellular electrophysiology study, both cell and brain slice numbers were equal to or higher than 5, although animal number N = 3–8. The exact group size for each experimental group is stated in each figure legend. Mice were 8–12 weeks of age at the study onset, included equal numbers of both males and females, ranged in weight from 20 to 30 g, and housed under specific pathogen free conditions (5 mice per cage) in a control environment at 20 ± 2 °C and humidity 50–60%. Mice were exposed to a 12-h light/dark cycle (8:00 AM–8:00 PM) with free access to a standard chow diet and drinking water.

### In vitro electrophysiology

Electrophysiological methods have been reported previously^10^. Briefly: GAD67-GFP transgenic mice of 6–10 weeks-old were anesthetized using phenobarbital sodium and decapitated. Coronal frontal slices (400 µm) were cut on a DTK-1000 tissue slicer (DTK, Japan) in 4 °C cutting aCSF (110 mM choline chloride, 7 mM MgSO_4_, 2.5 mM KCl, 1.25 mM NaH_2_PO_4_, 25 mM NaHCO_3_, 25 mM d-glucose, 11.6 mM sodium ascorbate, 3.1 mM sodium pyruvate, 0.5 mM CaCl_2_), allowed to recover for 30 min at 32 °C, then transferred to a holding chamber at room temperature in aCSF (127 mM NaCl, 2.5 mM KCl, 1.25 mM NaH_2_PO_4_, 25 mM NaHCO_3_, 25 mM d-glucose, 2 mM CaCl_2_, 1 mM MgCl_2_). Recording started at least 1 h after recovery. All animals were randomly used for all experiments. Recordings were made on an Olympus microscope (BX51WI) with a 40X water-immersion objective. Recordings were made from layer 2/3 of the AI. For evoked NMDAR EPSCs and sEPSCs/sIPSCs recordings, recording pipettes (4–8 MΩ) were filled with (in mM): 125 CsMeSO_4_, 5 NaCl, 1.1 EGTA, 10 HEPES, 0.3 Na_2_GTP, 4 Mg-ATP, 5 QX-314. For current clamp recordings, recording pipettes were filled with (in mM):128 K-Gluconate, 10 NaCl, 2 MgCl_2_, 0.5 EGTA, 10 HEPES, 4 Na_2_ATP, and 0.4 Na_2_GTP. Evoked AMPAR EPSPs were obtained at near resting membrane potentials in normal aCSF. To record evoked NMDAR EPSCs, neurons were held at − 60 mV in the presence of Mg^2+^ (0.5 mM), AMPAR blocker (NBQX, 10 µM), GABA_A_R blocker (picrotoxin, 50 µM) and GluN2B-NMDAR antagonist (Pip18, 1 µM) in the perfusion solution. To record sEPSCs and sIPSCs, neurons were held at − 60 mV or + 10 mV respectively in the normal aCSF. To obtain spontaneous spike firing in whole-cell configuration, most recorded neurons were held at − 45 ± 5 mV, via injection of current if necessary. Signals were acquired at a sampling rate of 10 kHz and filtered at 2 kHz. NMDAR EPSCs were analyzed off-line using Clampfit software (MDS Analytical Technologies), while sEPSC/sIPSCs and spontaneous spike firing were analyzed using Mini analysis (Synaptosoft).

### Osmotic pump implantation and hearing lesion

Mice were anesthetized with isoflurane (~ 1% in a gas mixture) in a stereotaxic apparatus. A craniotomy was made over the lateral ventricle (AP: − 0.5; ML: − 1.0; DV: 2.0) with a scalpel. Then a hole was drilled through the skull. To house the osmotic pump, a subcutaneous pouch was created gently on the back of a mouse by separating skin from the musculature with rounded-tip scissors. A cannula was slowly lowered until the pedestal touched the skull and then the pedestal was sealed to the skull with surgical glue. On the next day, the mouse was anesthetized with isoflurane (~ 1% in a gas mixture) in a sound attenuation chamber, where a continuous pure tone of 8 kHz was played at 114 dB for 2 h as noise exposure.

### In vivo electrophysiological recording and sensory stimuli

In vivo electrophysiological recording and sensory stimuli methods have been described previously^67^. In this study, two anesthetics were used to eliminate potential influence of anesthetics. A total of 242 cells/17 mice were recorded under ketamine (100 mg/kg) and xylazine (10 mg/kg) anesthesia, and another 133 cells/6 mice were recorded under 1% isoflurane anesthesia. During recording studys, mice were placed on a homoeothermic heating pad at 36.5 °C in a sound attenuation chamber. The head was secured with a custom eye-clamped head-holder that left ears under speaker. Following deflection of the temporal muscle, exposure of the right auditory cortex, and removal of the dura mater. The right auditory cortex was exposed and kept under layer of silicone oil to prevent desiccation. The extracellular signal was obtained using a TDT (Tucker Davis Technologies) amplifier (Medusa RA16PA) connected to TDT RX5 hardware using TDT software (OpenEx) running on a Windows 10 computer. Neural responses were recorded using 32 channel (4 × 8 configuration) silicon probes (neuroNexus) at layer2/3 of the right AI. Their responses to 25 ms tone pips of 51 frequencies (4 to 75 kHz) and 8 sound pressure levels (0–70 dB SPL, 10 dB steps) were recorded to separate spontaneous/evoke spikes and reconstruct the frequency-intensity receptive field (RF). After 20 min of baseline recording, M-8324 was infused into lateral ventricle at 0.5 µL/min for 10 min. Data analysis was done off-line with custom MATLAB programs^67^.

### Gap detection

In vivo electrophysiological recording and sensory stimuli methods have been described^68^. Briefly, mice were put in a small box with pressure sensor individually. The pressure changes for every startle stimulus were recorded. The gap was presented 50 ms between six different frequencies (5, 7, 10, 14, 20, 28 kHz) at 75 dB and the startle stimulus (white noise, 105 dB, 20 ms). After a 3 min acclimation period, 150 gap trials and 150 no-gap trials under six frequencies were performed randomly for each mouse. And calculate the pressure changes ratio of gap trials and no gap trials.

### Immunofluorescence staining and image analysis

Mice were perfused under deep anesthesia with ice-cold phosphate-buffered saline (PBS) followed by 4% paraformaldehyde (PFA). Brains were removed and fixed in PFA overnight at 4 °C, equilibrated in 30% sucrose. Coronal sections (20 µm in thickness) were cutting by cryostat microtome (Leica CM1950) and collected on gelatinized glass slides. After air drying, sections were washed in PBS and blocked with 0.3% Triton-X (Sigma), 10% goat sera (Boster) in PBS at room temperature for 1 h. And incubated with primary antibodies (PV polyclonal antibody, ab11427, Abcam) overnight at 4 °C. The secondary antibodies conjugated with Alexa Fluor 488 were incubated for 1 h at room temperature to enable fluorescent detection. After rinsing with PBS, the sections were mounted with fluorescence mounting medium and viewed under the confocal microscope (Nikon A1). All the images were taken on the same day using the same parameters/setting. PV staining analysis was performed using Image J (NIH image). PV cell density was counted manually and corrected for the size of layer 2/3 in AI.

### Statistical analysis

Studies were designed to employ exposure and treatment groups of equal size, randomized to experimental conditions, and with experimenters blind to the analysis. Statistical analysis was performed only on studies with group size equal to or larger than 5. Group sizes represent the number of independent values, with statistical analysis using these values, technical replicates were not treated as independent values.

Individual recorded neurons were sorted using Plexon Offline Sorter. Sorted spikes were analyzed using custom-made Matlab software^69^ and Neuro Explorer. We made a scatter diagram based on the waveforms of all recorded neurons. The half-width of action potential waveform as abscissa, and the peak to trough time as ordinate. According to the clustering of scatter plots, all neurons are classified into two groups based on waveforms. One group with narrower waveforms is considered as inhibitory neurons, the other group with wider waveforms is considered as excitatory neurons. We computed the Pearson correlation coefficient of evoked spike rate in excitatory and inhibitory neurons between 90 and 100 min after M-8324 infusion in each mouse as trial to trail reliability index^13^. Statistical significances were calculated using unpaired two-tailed Student’s *t* test or one-way, two-way ANOVA with the Bonferroni's post hoc test, while post hoc tests were performed only if F achieved *P* < 0.05. All graphing and statistical analyses were carried out using GraphPad Prism software (Version 6, USA). Data are reported as mean ± SEM. Significance is noted as *P < 0.05, **P < 0.01.

## Supplementary information

Supplementary file1 (DOCX 566 kb)

## Data Availability

The data that support the findings of this study are available from the corresponding author upon reasonable request.
