# Supplementary material for: Increasing endogenous activity of NMDARs on GABAergic neurons increases inhibition, alters sensory processing and prevents noise-induced tinnitus
Source: Sci Rep. 2020 Jul 20;10:11969. doi: 10.1038/s41598-020-68652-5 (PMC7371882; doi:10.1038/s41598-020-68652-5)
Supplement: Supplementary file 1 — Supplementary file1 (DOCX 566 kb) [file 41598_2020_68652_MOESM1_ESM.docx]

**Increasing endogenous activity of NMDARs on GABAergic neurons increases inhibition, alters sensory processing and prevents noise-induced tinnitus**

Di Deng^1^, Samer Masri^2^, Lulu Yao^1, 3^, Xiaoyan Ma^1^, Xuebing Cao^1^, Sungchil Yang^4^, Shaowen Bao^2^ and Qiang Zhou^1, 5, *^

1, School of Chemical Biology and Biotechnology, Peking University Shenzhen Graduate School, Shenzhen, China

2, Department of Physiology, University of Arizona, Tucson, AZ 85724, USA.

3, South China Research Center for Acupuncture and Moxibustion, Medical College of Acu-Moxi and Rehabilitation, Guangzhou University of Chinese Medicine, Guangzhou, China;

4, Department of Biomedical Sciences, City University of Hong Kong, Kowloon, Hong Kong.

5, State key laboratory of chemical oncogenomics, Peking University Shenzhen Graduate School, Shenzhen, China.

*, correspondence at zhouqiang@pkusz.edu.cn


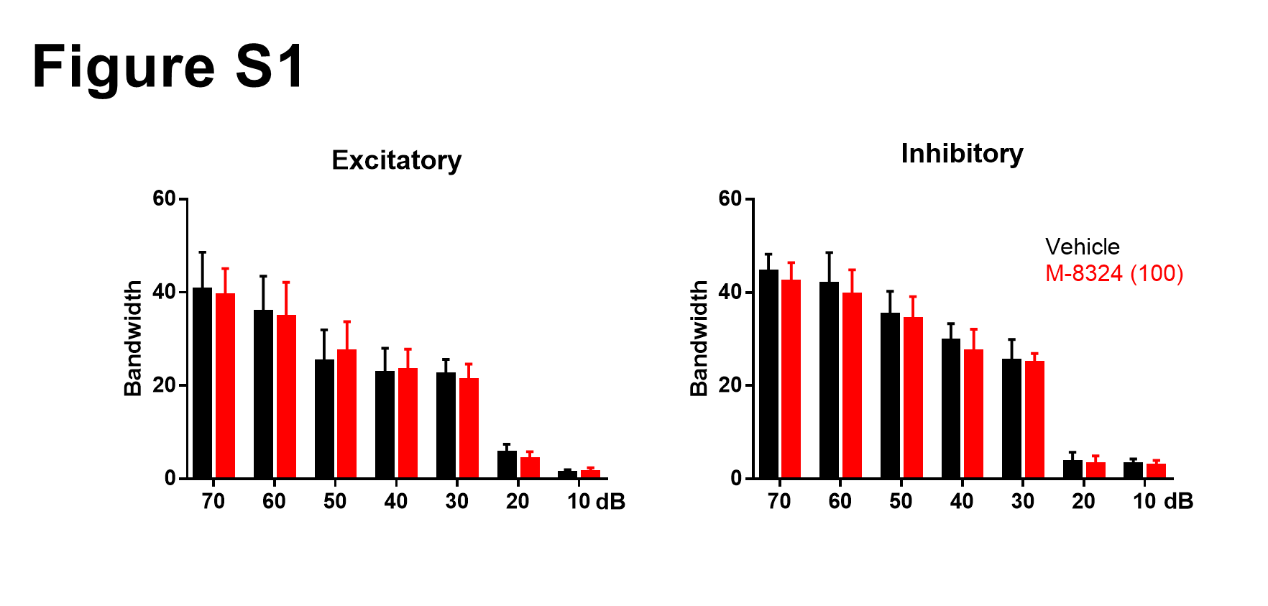


**Figure S1. M-8324 does not alter the bandwidth of tuning curves.** Quantitative analysis of spike tonal receptive fields of all recorded neurons. Tonal receptive field bandwidth was calculated from 10 to 70 dB in excitatory neurons (A), and inhibitory neurons (B). There were no differences between the vehicle and M-8324 (100 μM) groups. N = 8 and 9 mice, respectively, for vehicle and M-8324 (100 μM). Data are presented as the mean ± SEM.


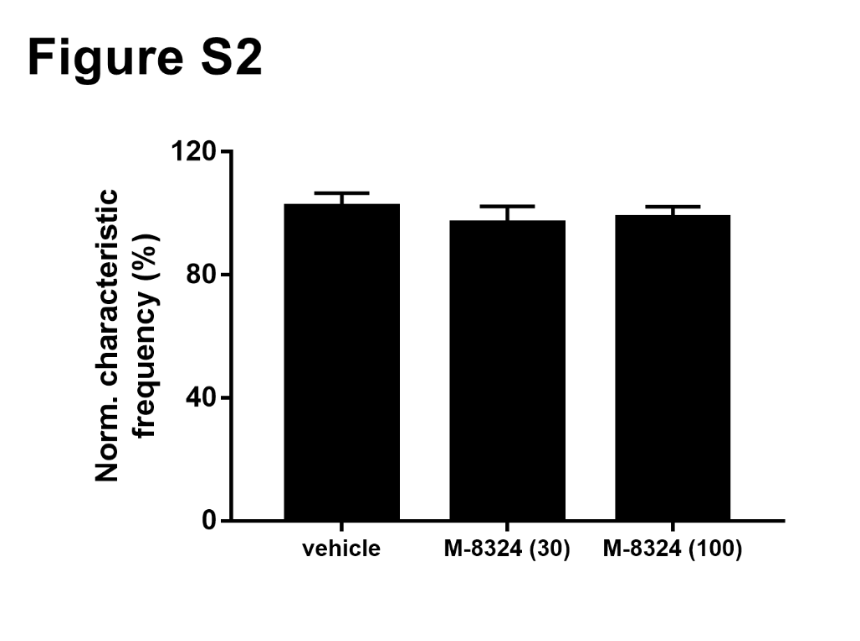


**Figure S2. M-8324 does not alter A1 characteristic frequency.** Quantitative analysis of spike tonal receptive fields of all recorded neurons with normalized characteristic frequency in the tonal receptive field. There were no differences among vehicle, M-8324 (30 μM) and M-8324 (100 μM) groups. N = 8 mice (vehicle), N = 6 mice (M-8324 30 μM), N = 9 mice (M-8324 100 μM). Data are presented as the mean ± SEM.


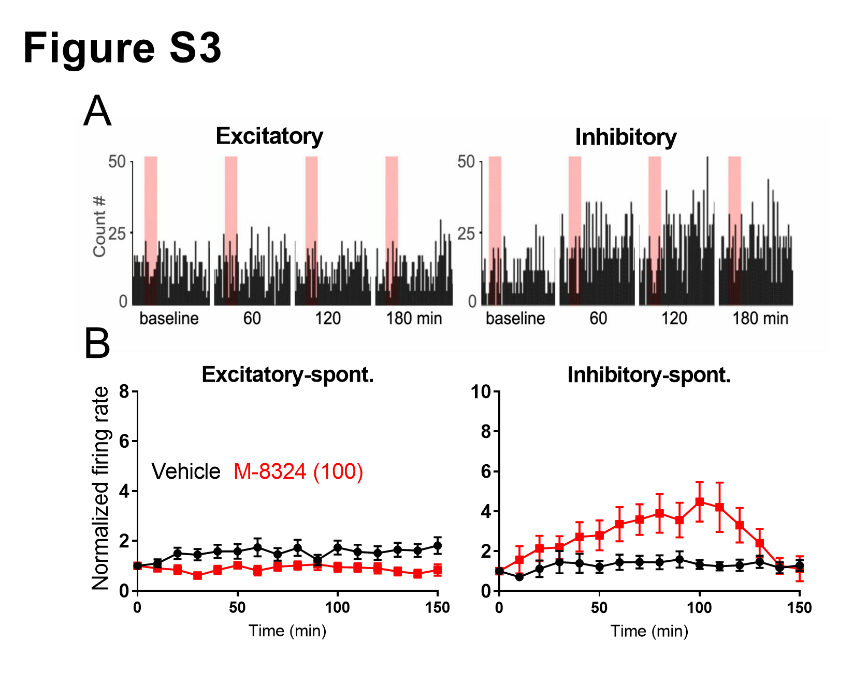


**Figure S3. M-8324 has similar impact on neurons with no response to sound.** (A) Histogram sample showing spike rate before and after M-8324 infusion. (B) Population and time course of changes in spontaneous spiking in excitatory and inhibitory neurons before and after infusion of M-8324. For excitatory cells, N = 19 cells/5 mice (vehicle), 18 cells/6 mice (M-8324 100 μM). For inhibitory neurons, N = 7 cells/5 mice (vehicle), 6 cells/6 mice (M-8324 100 μM). Data are presented as the mean ± SEM. * represent significance versus vehicle group.

**
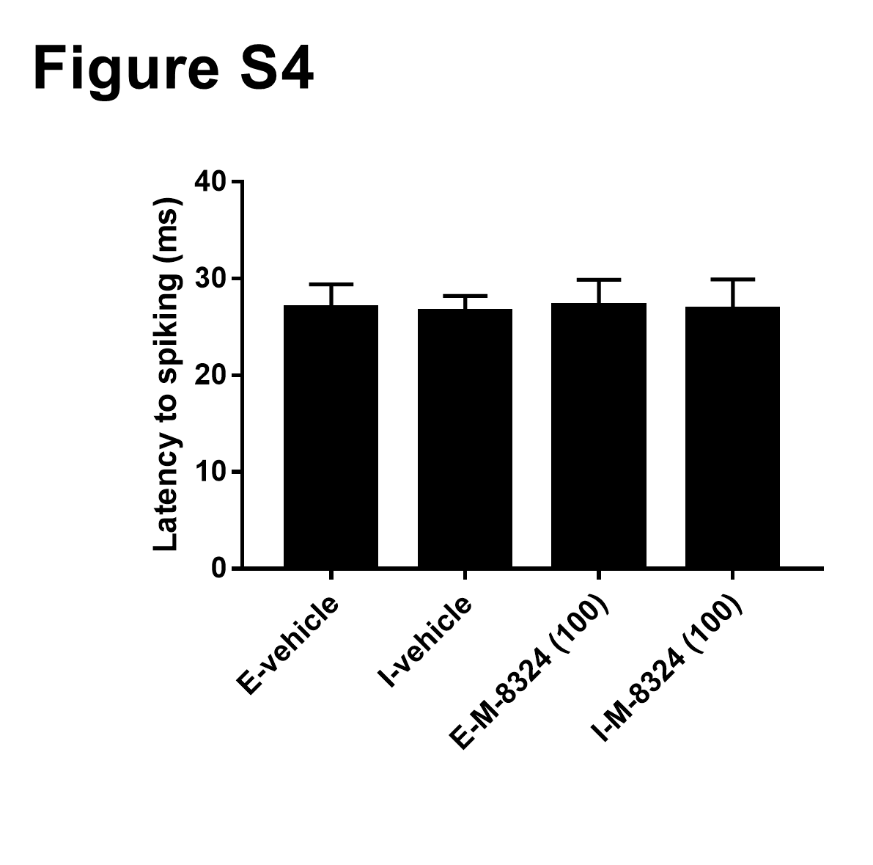

Figure S4. M-8324 does not alter the** **onset latency of first tone-evoked spike.** Analysis of onset latency of the first tone-evoked spike. There was no difference between excitatory and inhibitory neurons in vehicle group and M-8324 (100 μM) group. N = 95 cells/8 mice (E-vehicle), N = 28 cells/8 mice (I-vehicle), N = 114 cells/9 mice (E-M-8324 100 μM), N = 46 cells/9 mice for (I-M-8324 100 μM). Data are presented as the mean ± SEM.

**Supporting table 1. Summary of tested parameters.**

|  | 30 μM M-8324 | 100 μM M-8324 |
| --- | --- | --- |
| Tuning curve BW | no change | no change |
| Tuning curve CF | no change | no change |
| Onset Latency (evoked) | no change | no change |
| Spike Freq (E) | decreased | Decreased |
| Spike Freq (I) | increased | Increased |
| SNR (E) | no change | Increased |
| SNR (I) | no change | no change |
| E/I ratio | decreased | Decreased |
| Reliability | no change | Increased |
| Spike Duration (evoked) | no change | Decreased |

Dose-dependence of the examined parameters. BW, bandwidth; CF, characteristic frequency. Parameters were measured in excitatory neurons if not specified.
